# Diarylboron‐Based Asymmetric Red‐Emitting Ir(III) Complex for Solution‐Processed Phosphorescent Organic Light‐Emitting Diode with External Quantum Efficiency above 28%

**DOI:** 10.1002/advs.201800950

**Published:** 2018-07-18

**Authors:** Xiaolong Yang, Haoran Guo, Boao Liu, Jiang Zhao, Guijiang Zhou, Zhaoxin Wu, Wai‐Yeung Wong

*Adv. Sci*. **2018**, *5*, 1701067

The Acknowledgements originally published for this article were incomplete. The following information should be added:

W.‐Y.W. also thanks the financial support from the Hong Kong Polytechnic University (1‐ZE1C) and Ms. Clarea Au for the Endowed Professorship (847S).

